# Focus Group Findings of Trial Participants in a Caregiver Psychosocial Intervention: Savvy Caregiver Program

**DOI:** 10.1093/geroni/igab046.3607

**Published:** 2021-12-17

**Authors:** Maria Aranda, Iris Aguilar, Rene Maldonado

**Affiliations:** University of Southern California, Los Angeles, California, United States

## Abstract

Previous work highlights the importance of sociobehavioral interventions to address dementia caregiving health and wellbeing outcomes. By empowering caregivers to become objective managers of their loved one’s illness, and own self-care, they are less likely to exhibit negative outcomes. We are conducting a mixed-method, randomized trial to test manualized, multi-family psychoeducational group interventions: Savvy Caregiver Express, and Savvy Caregiver Program. This poster describes the qualitative findings of four focus group interviews recently conducted to elucidate the study participation experiences of family caregivers enrolled in the parent study. Twenty-five racially and ethnically diverse participants (21 women, 4 men) caring for a family member with cognitive decline participated in focus group interviews conducted via videoconferencing methods. We captured more nuanced experiences from the perspective of study participants with regards to the caregiver interventions and their research participation. Interviews were conducted by trained research personnel, lasted 60-75 minutes, and followed an open-ended questioning route. Based on thematic analyses, we identified the following themes: 1) Changing one’s mindset: Seeing life through their shoes; 2) Getting information in one place; 3) Expanding the personal experience; 4) Fears and vulnerability; 5) Time constraints vs. wanting more; 6) Not everyone is at the same place; 7) Technology: It’s going to be part of our lives; and 8) Research: Not always in sync. Our findings indicate high satisfaction with most components of the program while specific recommendations were offered to improve the intervention and study experience such as tailoring materials to stage-specific needs.

